# Diversity and interactions among triatomine bugs, their blood feeding sources, gut microbiota and *Trypanosoma cruzi* in the Sierra Nevada de Santa Marta in Colombia

**DOI:** 10.1038/s41598-021-91783-2

**Published:** 2021-06-10

**Authors:** Claribel Murillo-Solano, Jaime López-Domínguez, Rafael Gongora, Andres Rojas-Gulloso, Jose Usme-Ciro, Erick Perdomo-Balaguera, Claudia Herrera, Gabriel Parra-Henao, Eric Dumonteil

**Affiliations:** 1 Department of Tropical Medicine, School of Public Health and Tropical Medicine, and Vector-Borne and Infectious Disease Research Center, Tulane University, 1440 Canal St., New Orleans, LA 70112 USA; 2 Centro de Investigación en Salud para el Trópico, Universidad Cooperativa de Colombia, Santa Marta, Colombia; 3 LADISER Inmunología y Biología Molecular, Facultad de Ciencias Químicas, Universidad Veracruzana, Orizaba, Veracruz México; 4 Doctorado en Ciencias Biomédicas. Centro de Investigaciones Biomédicas, Universidad Veracruzana, Xalapa, Veracruz México; 5 Secretaria de Salud Distrital, Santa Marta, Colombia; 6 Instituto Nacional de Salud, Bogotá, Colombia

**Keywords:** Parasitic infection, Molecular ecology

## Abstract

Chagas disease remains a major neglected disease in Colombia. We aimed to characterize *Trypanosoma cruzi* transmission networks in the Sierra Nevada de Santa Marta (SNSM) region, to shed light on disease ecology and help optimize control strategies. Triatomines were collected in rural communities and analyzed for blood feeding sources, parasite diversity and gut microbiota composition through a metagenomic and deep sequencing approach. *Triatoma dimidiata* predominated, followed by *Rhodnius prolixus, Triatoma maculata, Rhodnius pallescens, Panstrongylus geniculatus* and *Eratyrus cuspidatus.* Twenty-two species were identified as blood sources, resulting in an integrated transmission network with extensive connectivity among sylvatic and domestic host species. Only TcI parasites were detected, predominantly from TcIb but TcIa was also reported. The close relatedness of *T. cruzi* strains further supported the lack of separate transmission cycles according to habitats or triatomine species. Triatomine microbiota varied according to species, developmental stage and *T. cruzi* infection. Bacterial families correlated with the presence/absence of *T. cruzi* were identified. In conclusion, we identified a domestic transmission cycle encompassing multiple vector species and tightly connected with sylvatic hosts in the SNSM region, rather than an isolated domestic transmission cycle. Therefore, integrated interventions targeting all vector species and their contact with humans should be considered.

## Introduction

Chagas disease remains a major neglected disease in many American countries, including Colombia, despite intensive efforts at vector control to reduce transmission to humans. There are an estimated 6–8 million persons in the Americas with Chagas disease, causing a burden of 29,000,000 DALYs and a health care cost of $24.73 billion^1–3^. In Colombia, there are over 400,000 cases of *T. cruzi* infection, and over 5000 new cases per year due to vectorial transmission^4^.

In particular, the Sierra Nevada de Santa Marta (SNSM) region in northeastern Colombia is considered as a hyper-endemic region, with a human seroprevalence of 16–57% in some communities^5–8^. Such a high prevalence is associated with complex *T. cruzi* transmission cycles that involve several species of triatomines, including *Triatoma dimidiata, Triatoma maculata, Rhodnius prolixus, Rhodnius pallescens, Rhodnius pictipes, Rhodnius neivai, Panstrongylus geniculatus*, *Panstrongylus rufotuberculatus*, and *Eratyrus cuspidatus* and an extensive diversity of potential mammalian hosts^5,6,9–11^. These vector species show diverse levels of adaptation to the domestic environment, with *R. prolixus* and *T. dimidiata* able to colonize houses, and possibly *R. pallescens*, while *T. maculata* is mostly found in peridomiciles, often associated with bird nests, and *E. cuspidatus* is mostly intrusive and more rarely observed^6,12,13^. Nonetheless, several of these species have been found to frequently blood feed on humans^5,14^, and can contribute to the risk of human infection^15^.

Furthermore, additional layers of complexity are added to these *T. cruzi* transmission cycles when considering parasite diversity, and its potential interactions with triatomine gut microbiota, which may modulate parasite development^16–18^. Indeed, of *T. cruzi* as a species has been divided into seven discrete typing units (DTUs TcI to TcVI and Tcbat)^19,20^, which represent highly stable near-clade parasite populations^21–23^. This genetic structure is thought to be maintained by the mostly clonal propagation of *T. cruzi*^21,24^, although recent evidences indicate the occurrence of sexual reproduction and recombination associated with a high level of inbreeding, at least within TcI DTU^25,26^. In Northeastern Colombia, TcI largely predominates, although some non-TcI DTUs have also been reported^5^. Within TcI, TcIa, TcIb and TcId subgroups^27,28^ have been identified by PCR genotyping in patients, vectors and some reservoir hosts, and these initial studies suggested some overlap among sylvatic and domestic parasite transmission cycles^6,29^. A better understanding of these potential overlaps and the contribution of different triatomine species to human transmission is critical to optimize vector control strategies and ensure their efficacy^30^.

While some of these aspects have been investigated in an isolated manner in previous studies as detailed above, their integration to understand transmission cycles and Chagas disease ecology has been limited. Furthermore, new approaches based on deep sequencing of selected markers allow for an unprecedented depth of analysis to disentangle feeding and parasite transmission cycles from different vectors and the multiple interactions shaping *T. cruzi* parasite diversity^31,32^. In this study, we aimed to characterize *T. cruzi* transmission networks based on the identification of vertebrate feeding host diversity as well as parasite and microbial diversity in triatomines from the SNSM region in northwestern Colombia, to shed light on Chagas disease ecology in the region and help optimize current control strategies.

## Results

### Triatomine diversity

**Figure 1:**
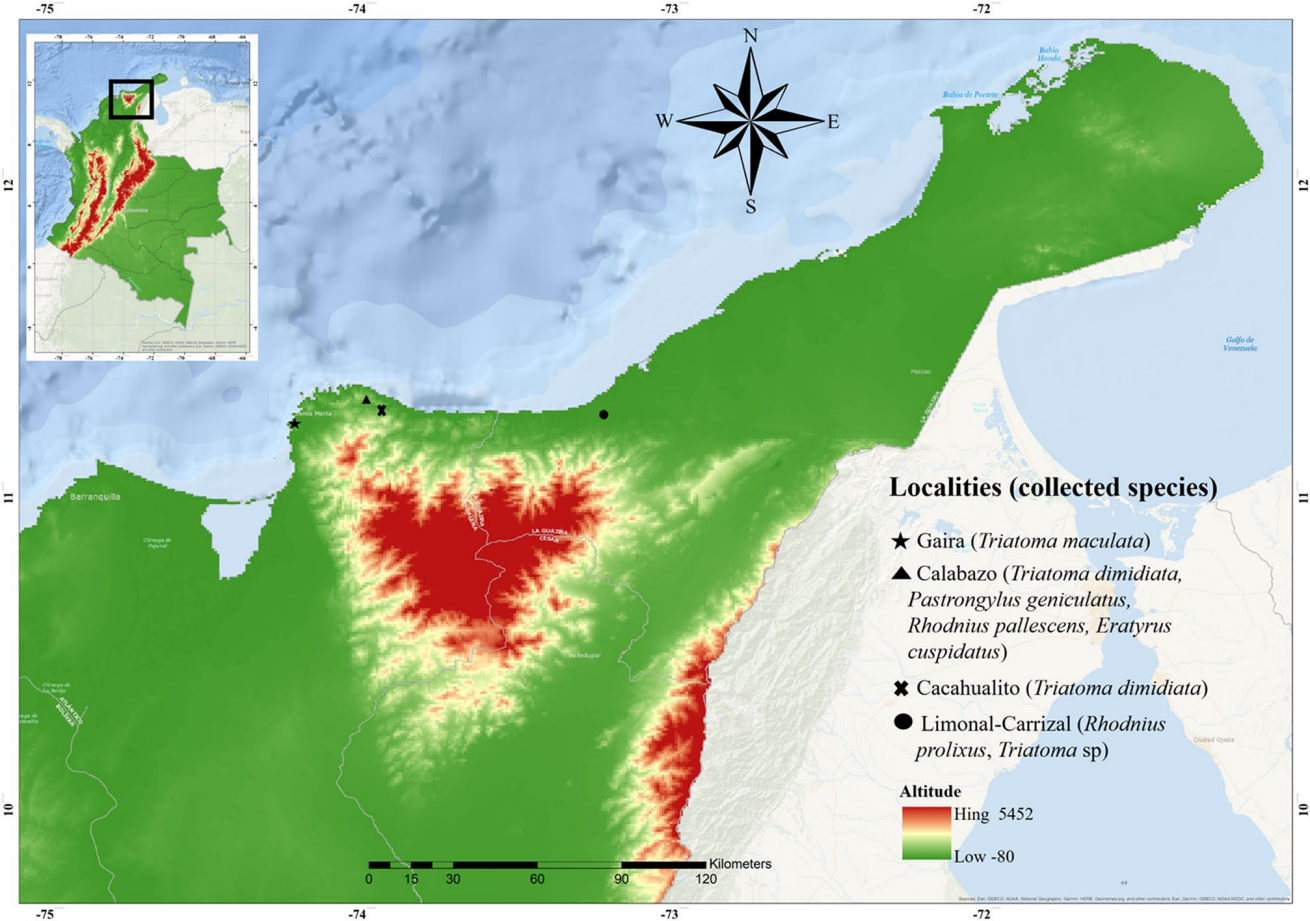
Map of the study area. Triatomine collections were performed in the indicated localities of the SNSM region. Insert: map of Colombia. This map was created in QGIS 3.4 (https://www.qgis.org/en/site/).

Our study included the analysis of a convenience sample of 42 bugs collected inside and around rural houses (Table 1) in Guajira and Magdalena departments in the SNSM in northeastern Colombia (Fig. 1). Analysis of ITS-2 and Cyt B sequences confirmed the morphological identification of species for adults and allowed the identification of the nymphs. The most abundant species was *T. dimidiata*, followed by *R. prolixus, T. maculata, R. pallescens, P. geniculatus* and *E. cuspidatus* (Table 1).

**Table 1:** Triatomine species in the study area.

Species	Number (%)	Sex/stages^a^	Habitat
*T. dimidiata*	17 (40.5)	6 F, 4 M, 6 N	Palm trees, 5–20 m from houses
*R. prolixus*	12 (28.6)	2 F, 1 M, 4 N	Intradomicile
*T. maculata*	6 (14.3)	1 F, 5 M	Peridomicile, 5–10 m from houses
*R. pallescens*	3 (7.1)	2 M, 1 N	Palm trees, 5–20 m from houses
*P. geniculatus*	3 (7.1)	2 F, 1 M	Peridomicile, 5–10 m from houses
*E. cuspidatus*	1 (2.4)	1 M	Peridomicile, 2 m from house

*F* female, *M* male, *N* nymph.

^a^The sex of six adult bugs could not be determined (one *T. dimidiata* and five* R. prolixus*).

### Triatomine feeding profiles

**Figure 2:**
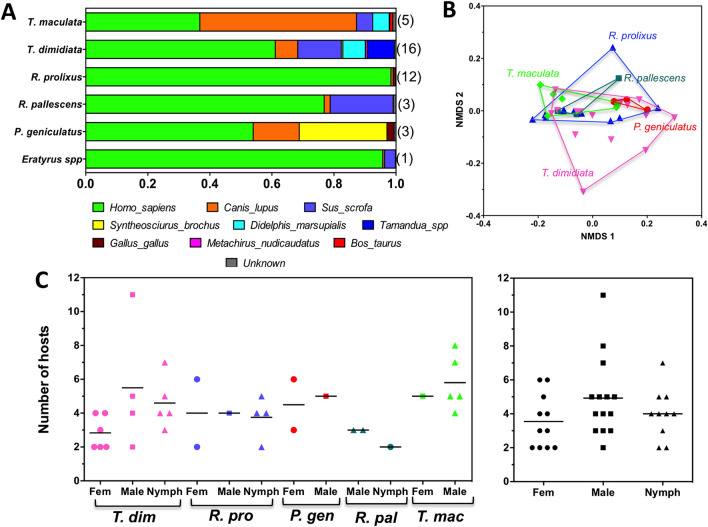
Triatomine feeding profiles. (**A**) Feeding profile of the indicated triatomine species. Vertebrate feeding hosts are color coded as indicated. Numbers in parenthesis on the right indicate sample size. Note the different feeding proportion on humans. (**B**) NMDS analysis of feeding profiles among triatomine species. PERMANOVA, P = 0.027 (*R. pallescens, P. geniculatus* and *E. cuspidatus* were excluded from the statistical analysis due to the limited sample size for these species). (**C**) Number of concomitant feeding hosts species detected in single bugs according to triatomine species (left panel) and according to sex/developmental stage (right panel). *T. dim: T. dimidiata; R. pro: R. prolixus; P. gen: P. geniculatus; R. pal: R. pallescens; T. mac: T. maculata.*

Analysis of blood meal sources based on 12 s RNA sequences indicated that bugs fed on a wide diversity of vertebrate host species, with a total of 22 species identified as blood sources, covering mammals, birds, reptiles, and amphibians. However, most blood meals focused on a more limited diversity of vertebrate species, including domestic (human, dog, pig) and sylvatic species (squirrel, opossum, porcupine, or anteater) (Fig. 2A). Notably, while the triatomine species collected in this study shared the same macrohabitat and were found in sympatry, their feeding profiles varied significantly (Fig. 2A,B). *R. prolixus* and *E. cuspidatus* presented the highest proportion of blood meals on humans (> 95%), followed by *R. pallescens, T. dimidiata*, and *P. geniculatus*. On the other hand, *T. maculata* was the species with the lowest proportion of blood meals on humans (30%). As noted before in other triatomine species, most bugs had fed on multiple host species, ranging from 2 to 11 species, and the number of feeding host was comparable among triatomine species (Fig. 2C). Overall, female bugs had fed on 3.5 ± 0.5 host species, males on 4.9 ± 0.6 host species and nymphs on 4.0 ± 0.5 host species (ANOVA F = 1.70, P = 0.19), indicating a similar host-switching behavior among these triatomines (Fig. 2C).

**Figure 3:**
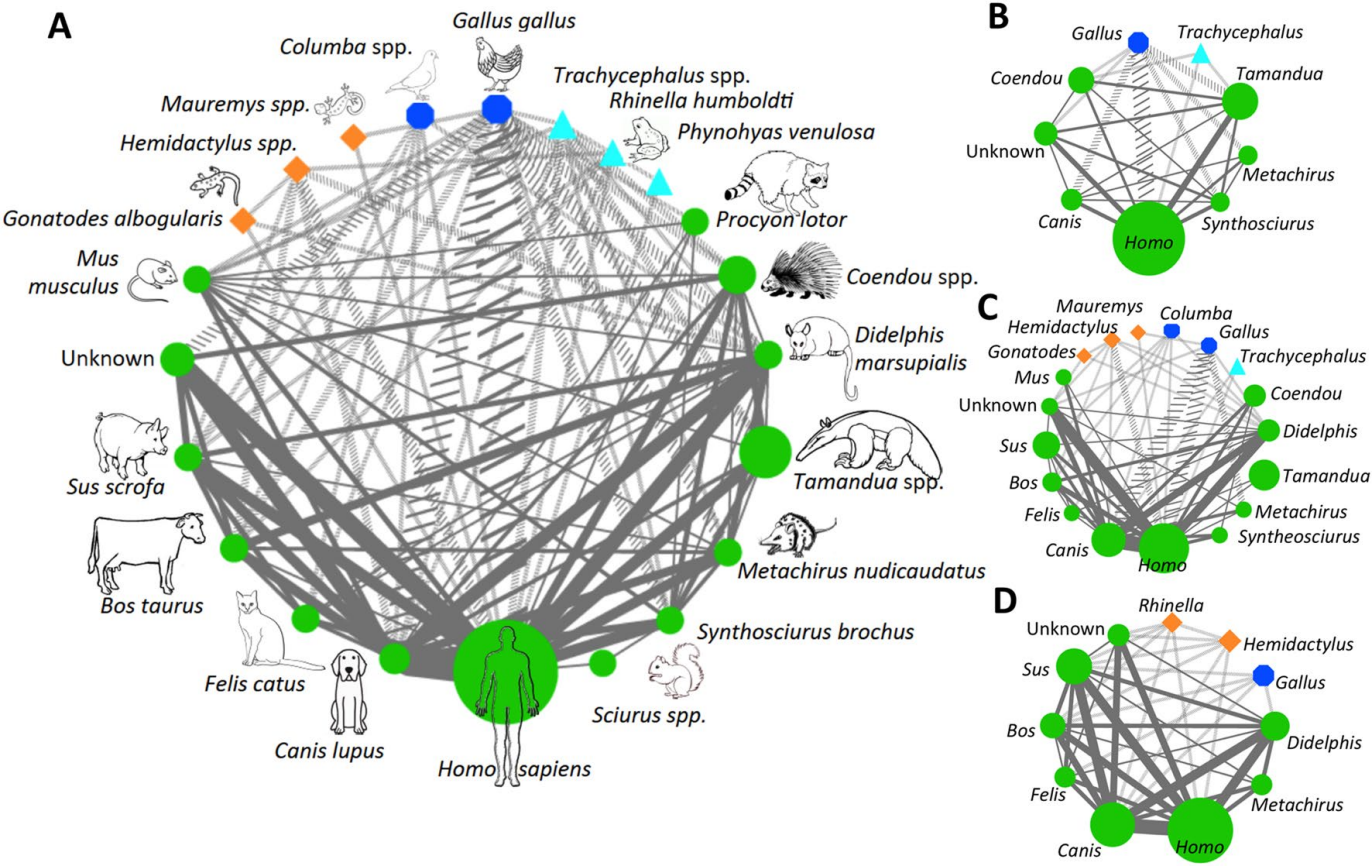
Triatomine feeding and parasite transmission networks. (**A**) Global triatomine feeding and *T. cruzi* transmission network in the SNSM region. This network integrates triatomine species diversity and their relative abundance, as well as the co-occurrence of host species in multiple blood meals from individual bugs, and triatomine blood feeding profiles. The size of vertebrate hosts nodes is proportional to the frequency of blood meals on each host species, and the width of the edges connecting the hosts is proportional to the frequency of their co-occurrence in single bug blood meals. Solid edges link mammalian hosts (green circles), which are competent hosts for *T. cruzi*, while dotted edges link non-competent hosts (amphibians: light blue triangles, birds: blue octagons, and reptiles: orange diamonds). (**B**–**D**) Feeding and parasite transmission networks for *R. prolixus*, *T. dimidiata* and *T. maculata*, respectively. For clarity, only the genus of hosts is indicated. Networks are based on data from 12, 17, and 6 bugs, respectively.

The co-occurrence of host species in multiple blood meals from individual bugs provides direct evidence of possible cross-species transmission pathways of *T. cruzi* parasite when occurring among mammalian species. This information together with the overall feeding profiles of bugs was used to build a parasite transmission network (Fig. 3A). As mentioned above, at least 22 vertebrate species were found to be involved in this network, emphasizing the ecological diversity of blood feeding hosts for triatomines in the region and the complexity of their interactions in *T. cruzi* transmission. Indeed, most host species in the network were well connected (as evidenced by a network density of 0.424), which provides extensive opportunities for cross-species transmission of *T. cruzi*. Importantly, there were extensive connections between domestic, synanthropic, and sylvatic mammalian species, suggesting that *T. cruzi* parasites infecting opossums, porcupines or anteaters could be transmitted to domestic animals and humans (and possibly vice-versa). A few hosts were somewhat isolated in the network, as indicated by a high network centralization statistic (0.633). Furthermore, multiple non-competent hosts including birds, amphibians and reptiles also served as blood sources and help maintain triatomine populations. Finally, an average network heterogeneity of 0.556 indicated the lack of hub nodes or dominant hosts in the network topology.

Nonetheless, analysis of triatomine species for which sample size was sufficient revealed that each was involved in very different networks. Indeed, *R. prolixus* network included the lowest diversity of feeding hosts, with only nine species, but these were much more connected as indicated by a high network density (0.750) and a low network centralization (0.321) (Fig. 3B). Humans were also a clear dominant host and network hub as indicated by a low network heterogeneity (0.248). On the other hand, *T. dimidiata* was involved in a more diverse network with 18 host species highlighting a more opportunistic feeding behavior (Fig. 3C). This resulted in hosts being somewhat less connected, as indicated by a lower network density (0.353) and a higher network centralization (0.662), and no clear network hubs or host dominance was detected (network heterogeneity of 0.678). Finally, *T. maculata* network topology was more similar to that of *R. prolixus*, although a high diversity of up to 11 host species was involved. These were well connected (network density 0.740 and network centralization 0.311) (Fig. 3D).

### *T. cruzi* infection

*Trypanosoma cruzi* infection was detected in 18/42 bugs (42.8%), corresponding to 1/3 (33.3%) in *P. geniculatus* and *R. pallescens*, 0/12 (0%) in *R. prolixus*, 10/17 (58.8%) in *T. dimidiata*, and 5/6 (83.3%) in *T. maculata*. Infection rate tended to be higher in adult bugs compared to nymphs (14/26, 53.8% *vs* 3/11, 27.2%, respectively, χ^2^ = 2.27, df = 1, P = 0.13). *T. rangeli* was also detected in one of the *R. pallescens* bug.

**Figure 4:**
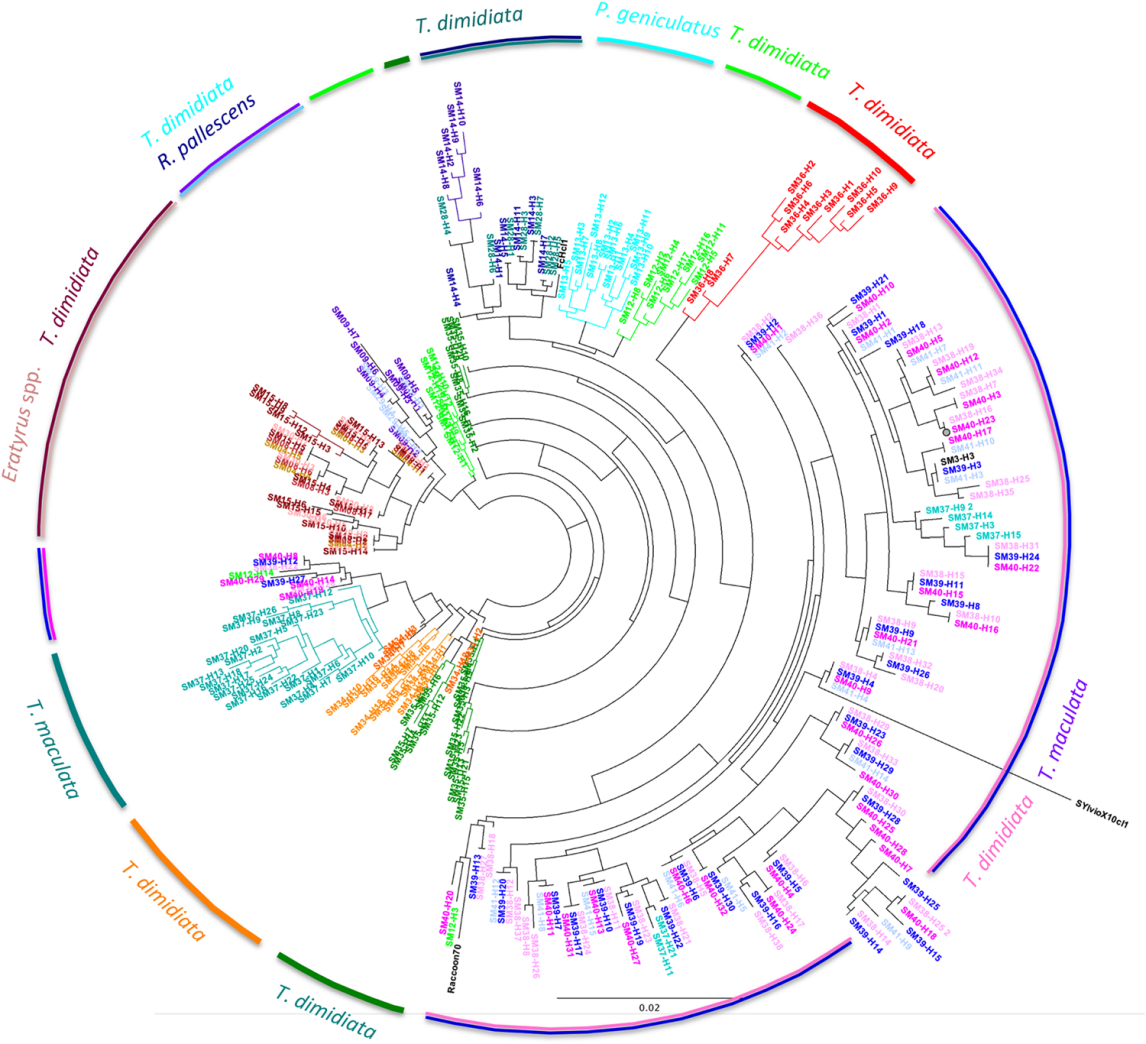
Phylogenetic analysis of *T. cruzi* mini-exon diversity. A total of 287 mini-exon sequences are shown that are color-coded for each individual bug analyzed. The corresponding triatomine species are indicated in the outer ring. Reference sequences from TcIa (Raccoon70 strain), TcIb (FcHcl1 strain) and TcId (SylvioX10 strain) are also included in the tree.

*Trypanosoma cruzi* parasites infecting bugs were genotyped using the mini-exon marker, and sequences were obtained through deep sequencing. A total of 287 mini-exon sequences (of 453 bp in length) were obtained, corresponding to 5–38 haplotypes per bug, ranging in frequency from 0.5 to 52.3%. All sequences belonged to TcI DTU. Phylogenetic analysis of these sequences showed that unique clusters of sequences were detected in several bugs (Fig. 4). Such clusters of sequences were likely paralogous sequences derived from a single parasite strain/clone, given the multicopy nature of the mini-exon marker, and illustrated well the Russian-doll pattern previously described^22,33,34^. A few bugs presented sequences from more than one cluster, suggesting potential infection with more than one strain of *T. cruzi*. Several bugs also shared nearly identical or closely similar parasite sequence haplotypes, indicating that they were infected with closely similar parasite strains. Furthermore, no genetic clustering of parasite sequences according to vector species was observed, and similar parasite haplotypes could be found in multiple triatomine species. This observation strengthened the extensive overlap of *T. cruzi* transmission cycles among these vector species, as indicated in Fig. 3.

**Figure 5:**
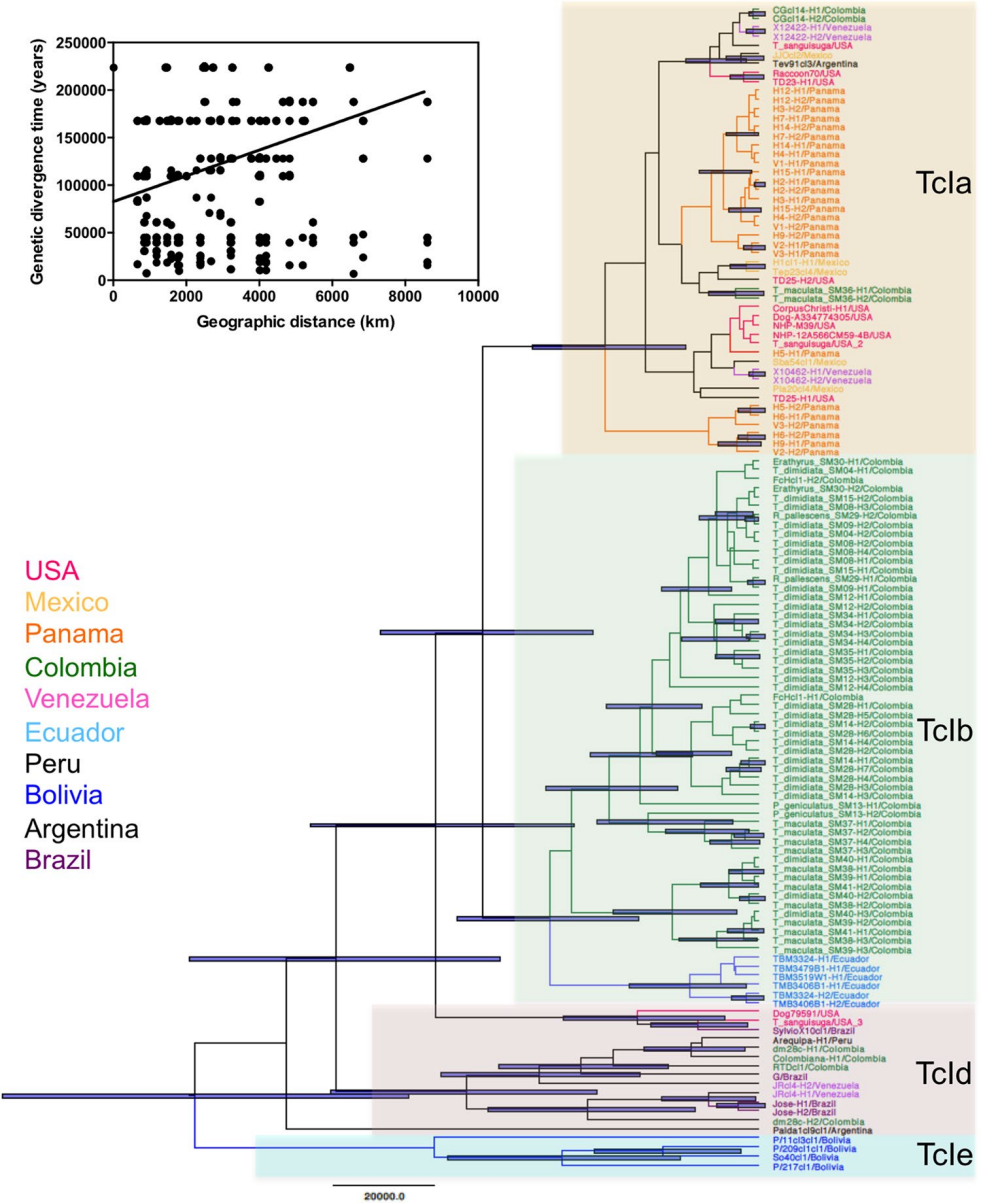
Phylogenetic analysis of TcI strains from northeastern Colombia. The best tree is shown based on Akaike information criteria, and *T. cruzi* sequences are color-coded according to their country of origin as indicated. The main TcI subgroups corresponding to TcIa, TcIb, TcId and TcIe are highlighted in different colors. Uncertainty in time of divergence is indicated for significant nodes (> 50% support). Insert: Mantel test indicating significant isolation by distance (R = 0.37, P = 0.0001).

To better understand the evolutionary history of TcI strains present in this region, we performed a further phylogenetic analysis including mini-exon sequences from additional strains from a more diverse geographic origin. Nearly all sequences from this study clustered within a large monophyletic group that included TcIb sequences from FcHcl1 reference strain as well as some sequences from Ecuador (Fig. 5). Thus, most parasites circulating among these bugs belonged to TcIb. One notable exception was one *T. maculata* bug, which harbored sequences that clustered with TcIa sequences from Panama, Venezuela, Mexico, and the USA, forming another well-defined monophyletic group. It is noteworthy that some other *T. cruzi* strains from Colombia clustered within TcIa and TcId subgroups, highlighting the diversity of TcI strains in this country. TcIa and TcIb parasites would have diverged about 55,000 years ago (± 20,000 years), and were clearly distinct from TcId parasites mostly found in Argentina and Brazil, which may include more than one cluster of sequences as it is a more diverse group^28^. TcIe included some of the oldest TcI parasite strains, mostly from Bolivia, which may represent the geographic origin of ancestral TcI DTU in the continent. Mantel test confirmed a significant isolation by distance of these TcI strains across the American continent (R = 0.37, P = 0.0001, Fig. 5 insert).

### Bacterial microbiota

**Figure 6:**
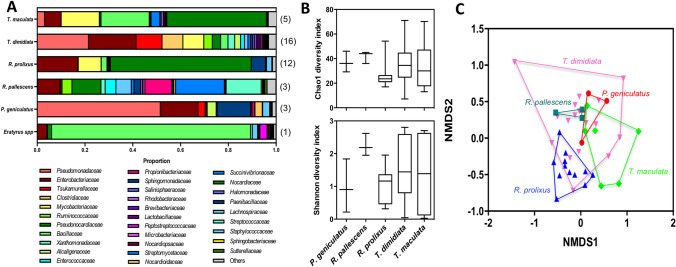
Composition of triatomine gut microbiota. (**A**) Proportion of bacterial families according to triatomine species. Taxonomic groups are color-coded as indicated and numbers in parenthesis on the right indicate sample size for each species. (**B**) Comparison of Chao1 (top panel) and Shannon (bottom panel) diversity indices among species. There were no significant differences among species (F = 0.82, P = 0.52 and F = 1.47, P = 0.23, respectively). (**C**) NMDS analysis of beta diversity among species. Microbiota composition was significantly different among species (PERMANOVA, F = 1.73, P = 0.006).

Analysis of the gut microbiota of these triatomines revealed an extensive diversity of bacteria as up to 77 families were identified, with proportions ranging from 0.5% up to over 85% (Fig. 6A). *Eratyrus cuspidatus* was excluded from further analysis due to its sample size. Comparison of alpha diversity within species indicated that the level of diversity was comparable among these triatomines, as indicated by Shannon and Chao1 diversity indices, although *R. pallescens* tended to present a somewhat higher diversity (Fig. 6B). However, beta diversity was significantly different among species (PERMANOVA, F = 1.73, P = 0.006), indicating species-specific differences in composition of the microbiota (Fig. 6C).

Thus, the abundance of some bacterial families varied among triatomine species. For example, *Pseudomonadaceae* and *Alcaligenaceae* were more abundant in *P. geniculatus* and *R. pallescens*, *Mycobacteriaceae* in *R. prolixus*, *Paenibacilaceae* in *R. prolixus* and *T. maculata*, *Clostridiaceae* in *R. pallescens* and *T. dimidiata*, among others (Supplementary Fig. S1). Noteworthy, *Wolbachia* was not identified in any of the bug analyzed.

**Figure 7:**
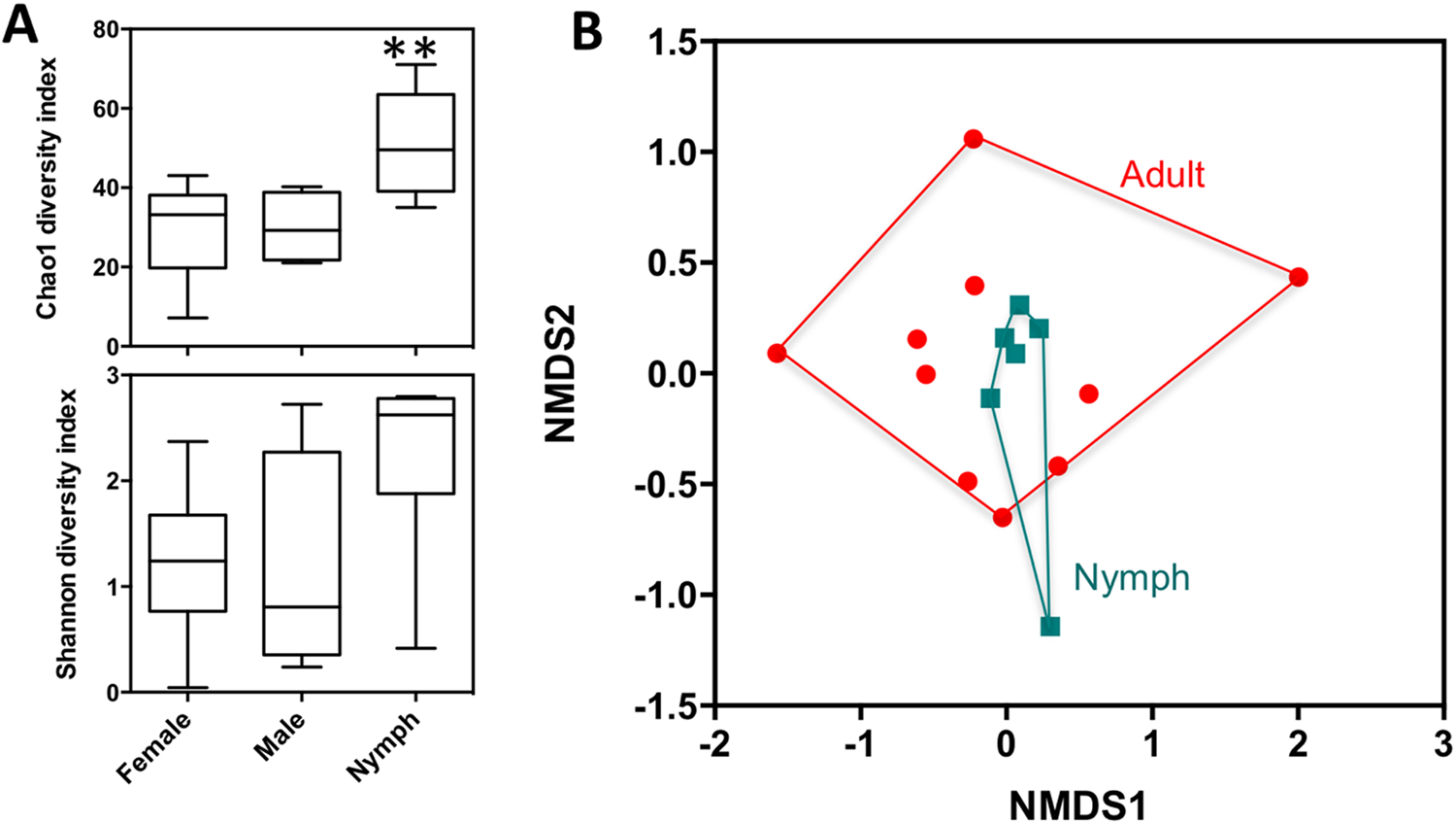
Microbiota diversity in *T. dimidiata*. (**A**) Alpha diversity assessed by Chao1 (top panel) and Shannon (Bottom panel) indices. Nymphs presented a significantly higher diversity based on Chao1 index (F = 5.82, P = 0.015), but not according to Shannon index (F = 2.64, P = 0.11). (**B**) NMDS analysis of beta diversity between adults and nymphs, indicating a significant difference in microbiota composition (PERMANOVA F = 1.84, P = 0.031).

We next focused on *T. dimidiata* microbiota, since we had a sufficient sample size to assess its diversity according to bug developmental stage/sex. Alpha diversity was significantly higher in nymphs compared to male and female adult bugs, as indicated by Chao1 index (F = 5.82, P = 0.015), but this did not reached statistical significance for Shannon index (F = 2.64, P = 0.11) (Fig. 7A). Multiple bacterial families such as *Mycobacteriaceae*, *Salinisphaeraceae*, *Methylobacteriaceae*, or *Nocardiospsaceae* were only detected in nymphs, and others such as *Pseudonocardiaceae*, *Streptomycetaceae*, or *Sphingomonadaceae* were more abundant in nymphs than in adult bugs. Beta diversity was also significantly different between adults and nymphs (PERMANOVA F = 1.84, P = 0.031), confirming differences in microbiota composition (Fig. 7B). These data indicated that microbiota composition changed during the development of the bugs, and that the emergence of adults was accompanied by a reduction in bacterial diversity in *T. dimidiata.*

**Figure 8:**
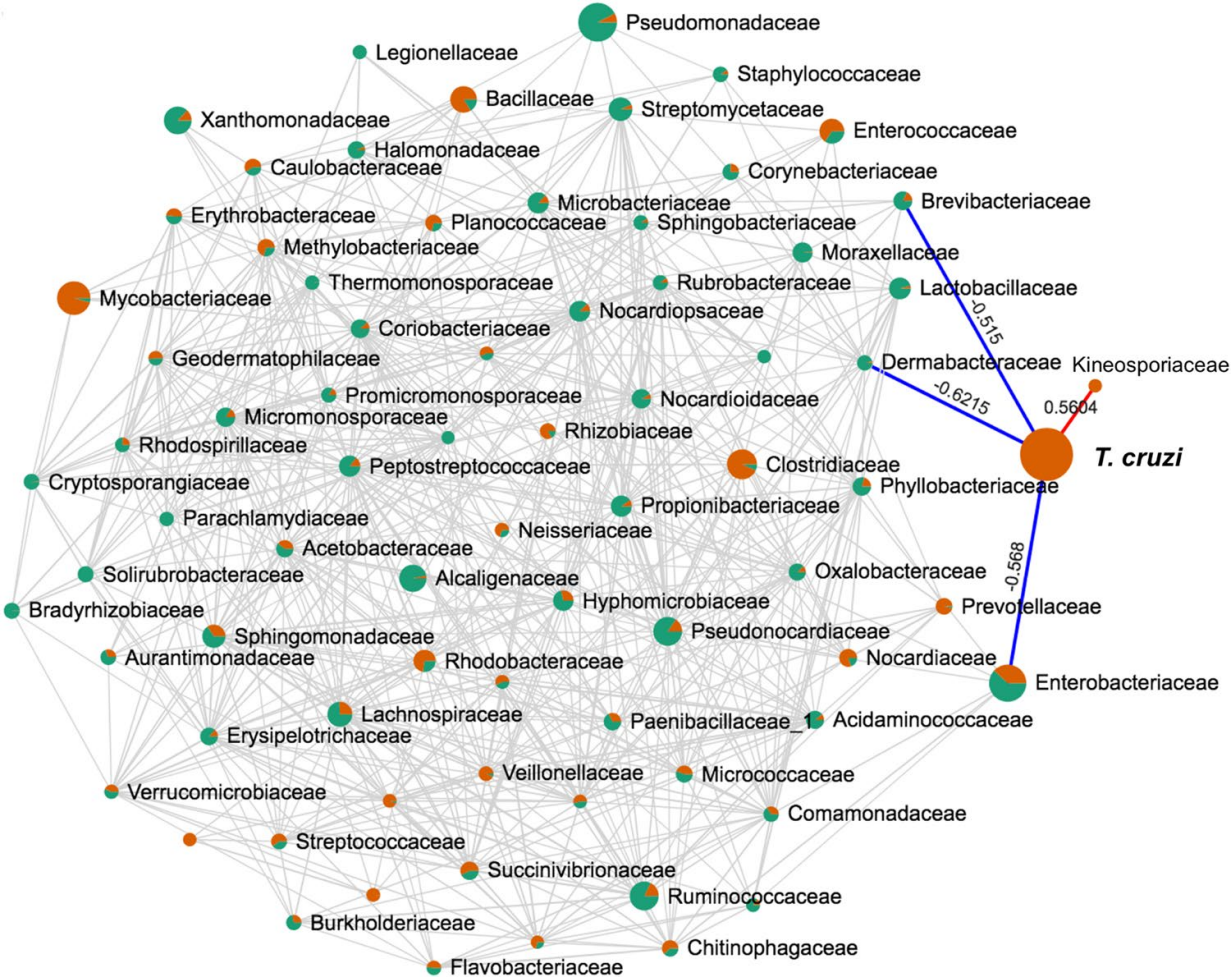
Correlation among bacterial families and *T. cruzi* in *T. dimidiata*. The network illustrates the diversity of bacterial families in *T. dimidiata*, indicated as nodes which size is proportional to the abundance of each family (except for *T. cruzi*). Green nodes indicate bacterial families found in uninfected bugs, and orange nodes indicate those found in *T. cruzi* infected bugs. Edges link nodes/families that are significantly correlated in the bugs (R > 0.35 and P < 0.05). *T. cruzi* infection was positively correlated (red edge) with the presence of Kineosporiaceae (R = 0.56), and negatively correlated (blue edges) with the presence of *Brevibacteriaceae* (R = − 0.52), *Dermabacteriaceae* (R = − 0.62) and *Enterobacteriaceae* (R = − 0.57).

Finally, we evaluated potential associations between *T. cruzi* parasites and the bacterial microbiota since they share the gut environment and may interact. We assessed pairwise correlations of *T. cruzi* with bacterial families, and found that *T. cruzi* infection was positively correlated with the presence of *Rhizobiaceae* (R = 0.39), and negatively correlated with the presence of *Burkhoderiaceae* (R = − 0.35), *Porphyromonadaceae* (R = − 0.39), and *Enterobacteriaceae* (R = − 0.51) (Supplementary Fig. S2). Further analysis focusing on *T. dimidiata* indicated that *T. cruzi* infection was positively correlated with the presence of *Kineosporiaceae* (R = 0.56), and negatively correlated with the presence of *Brevibacteriaceae* (R = − 0.52), *Dermabacteriaceae* (R = − 0.62) and *Enterobacteriaceae* (R = − 0.57) (Fig. 8). These data suggest potential interactions between *T. cruzi* and the bacterial community in triatomine guts, which may favor or limit *T. cruzi* development, and thus vectorial capacity.

## Discussion

Due to the complexity of *T. cruzi* transmission in the SNSM region in northeastern Colombia, we aimed to characterize parasite transmission networks based on the identification of vertebrate feeding host, parasite and microbial diversity in triatomine vectors. The diversity of vector species collected in our study is in general agreement with previous studies^15^. Thus, *T. dimidiata* and *R. prolixus* remain vectors of major concern in this region, but other species also need to be considered. Indeed, analysis of blood-feeding sources confirmed that all species collected fed on humans, as noted before^5,14^, although the frequency varied according to triatomine species. This highlights that all five species are epidemiologically relevant and need to be considered for vector control in the region.

Nonetheless, each triatomine species presented unique feeding profiles, which resulted in *T. dimidiata*, *T. maculata,* and *R. prolixus* being involved in very different feeding and parasite transmission networks. In addition, the predominance of adult bugs may indicate recent invasions rather than well-established colonies, except for *R. prolixus* which colonization is well documented. Thus, although these vectors species can be considered sympatric in the region, there are likely differences in their microhabitat leading to these differences in feeding profiles. For example, *T. dimidata* appeared as the most opportunistic vector, feeding on the broadest range of hosts that include a large diversity of domestic and sylvatic species, as seen before in southern Mexico^31^. These data suggest a high dispersal capacity of this vector between habitats, as also observed in the Andean region of Colombia^35^. On the other hand, *R. prolixus* had a more limited range of hosts, suggesting a more restricted microhabitat, in agreement with its frequent association with palm trees in multiple regions^15,36–38^. The high proportion of blood meals on humans is also in agreement with its role as a major vector species associated with frequent intrusions into human dwellings as well as its ability to colonize them^36,39^. Finally, *T. maculata* in the region appeared as a rather peridomestic vector feeding frequently on domestic hosts species, and further studies should help clarify the feeding profiles of *P. geniculatus* and *R. pallescens*. These data also illustrate well the sensitivity and usefulness of the metabarcoding and deep sequencing approach used here, which allowed the identification of a rather large diversity of feeding hosts from a limited number of bugs, as highlighted before^40,41^.

Despite the feeding specificities of each triatomine species, they also shared many host species, which resulted in an integrated transmission network showing extensive connectivity among sylvatic and domestic host species, including humans. This is a strong evidence of the presence of a single parasite transmission cycle encompassing all hosts and vector species in the region, rather than separate sylvatic and domestic cycles. Furthermore, no single mammalian species emerged as a key host/reservoir to maintain *T. cruzi* circulation, and parasite maintenance would be rather evenly spread over an important diversity of domestic and sylvatic mammals. In this context, manipulating host community structure to reduce parasite transmission to humans as proposed in other settings^42^ is unlikely to be very effective.

Analysis of *T. cruzi* sequences confirmed previous studies showing the presence of TcIa and TcIb in Northern Colombia^5,6,15^ and highlighted the predominance of TcIb in these vectors. TcIb was also found to predominate on nearby Margarita island as assessed by PCR^15^. The close relatedness of the *T. cruzi* strains, which clustered in a large TcIb monophyletic group, further support the conclusion that there are no separate transmission cycles according to domestic or sylvatic habitats, nor according to triatomine species. Interestingly, on a continental scale, we detected a significant genetic structuring of TcI parasites, as proposed before^22,43^, and the subgroups proposed before again formed clear clusters, except possibly TcId^27,28,44^. Also, the frequency of the respective TcI subgroups seems to vary according to the geographic region, with TcIa predominant in North America, and TcId and TcIe predominant in South America. In Colombia, a mixture of TcIa, TcIb and TcId subgroups is observed, and TcIb may be predominant, at least in this region. Remarkably, TcIe from Bolivia appeared as the most ancestral TcI, and may represent the source of TcI in the Americas with a more recent expansion and diversification in Central and North America. Further studies with additional strains and markers should help clarify how TcI subgroups have dispersed across the continent^45^.

Triatomines were also found to host species-specific microbiota, in general agreement with preliminary studies in *R. pallescens* and *T. maculata*^46^. Nonetheless, the microbiota of *R. pallescens* observed here was rather different from that of other populations in Panama, which also presented important differences among sites, raising questions on the stability of microbiota composition within species^47^. On the other hand, *T. dimidiata* microbiota included predominantly *Bacillales, Enterobacteriales* and *Clostridales* orders in Northern Colombia and Southern Mexico, although some minor differences could be observed^31^. Similarly, in *T. sanguisuga*, no differences in microbiota were observed among sites^32^. Importantly, *Wolbachia* was not detected in any of the species studied here, in agreement with multiple observations in triatomines^31,32,46,48^, but contrasting with its previous detection in *R. pallescens* from Panama^47^. As reported before for *R. prolixus*^48^, we also found evidence of ontogenic changes in microbiota in *T. dimidiata*, with an important reduction in diversity in adults compared to nymphs. This is in agreement with the key role of some endosymbionts in triatomine development^49^, but further studies are needed to identify the taxonomic groups involved and the nature of their interactions with the bugs. Finally, we confirmed important interactions of triatomine microbiota with *T. cruzi* infection, as observed in other triatomine species^32,50^. Thus, *Kineosporiaceae*, *Brevibacteriaceae*, *Dermabacteriaceae* and *Enterobacteriaceae* are emerging as taxonomic groups that may interact with *T. cruzi* in *T. dimidiata*. Importantly, Enterobacteriaceae have also been found to be negatively associated with *T. cruzi* infection in *Triatoma sanguisuga*^18^, suggesting that comparable interactions may occur across triatomine species. These observations raise the possibility of identifying bacterial species making triatomine bugs resistant to *T. cruzi* infection for vector control. Such approaches would be similar to *Wolbachia* and dengue virus in *Aedes*, or *Microsporidia* and *Plasmodium* in *Anopheles*^51,52^. A better understanding of these interactions in multiple triatomine species would be key to further evaluate the feasibility of such strategies.

Our study presents nonetheless some limitations, the main one due to the limited sample size and convenience sampling of bugs. Thus, further studies of additional bugs and sites would help strengthen our observations. Also, while the connection of the bugs collected here with sylvatic habitat is clearly evidenced by blood meals on sylvatic host species, further analyses of bugs from sylvatic habitats would be important to refine our understanding of parasite transmission cycles in this habitat.

In conclusion, we identified here an integrated *T. cruzi* transmission network based on vector feeding profiles, which evidenced extensive connectivity among sylvatic and domestic feeding hosts through multiple triatomine species. Limited parasite diversity lent further support to a single parasite transmission cycle encompassing all hosts and vector species in the region, rather than an isolated domestic transmission cycle. As a consequence, vector control focusing on a single species is unlikely to be effective, and no single reservoir host may be targeted. In this situation, integrated interventions targeting all vector species and their contact with humans should be considered, as discussed previously^30^. Finally, the limited parasite diversity further suggests important constraints, which may reflect a necessary adaptation of parasites having to be able to survive in a wide diversity of host species and interact with different bacterial communities in several vector species. Expanding such studies is needed to assess how host, vector, and parasite diversity are interconnected.

## Methods

### Study area

The SNSM is located in the extreme northwest of South America, north of Colombia, between latitude 10°–11° north and longitude 72°–74° west. It is made up of a mountainous group with a pyramidal shape and a triangular base of about 120 km on a side, extending from the Caribbean plain, at sea level, to a height of 5775 m on the Bolívar and Colón peaks. The study sites covered the northern slopes and associated lowlands within SNSM.

### Ethical statement

The study was approved by the Institutional Bioethics Committee of the Cooperative University of Colombia (UCC). Permission was obtained from homeowners to search their premises for bugs. All methods were carried out in accordance with relevant guidelines and regulations.

### Triatomine collection and DNA extraction

Triatomines were collected between January 2017 and August 2019 by a combination of community participation, manual searches and chicken-baited traps in several localities in the Guajira and Magdalena departments in the SNSM (Fig. 1). The sampling was done in the communities of Gaira (urban, located in the city of Santa Marta), Cacahualito and Calabazo in the rural area of Santa Marta, near the natural national Tayrona park, and Limonal-Carrizal (rural area) in the Guajira department. Two environments were covered: intradomicile and peridomicile. The dwellings included in the study were a convenience sample from a pre-existing list of houses with triatomine infestation from the Santa Marta's health office.

Three triatomine collection methods were used: active search, passive search and community search. The active search of adults and nymphs was carried out inside the dwellings (intra-domicile) in dark places, under furniture, beds, mats, and mattresses, inside the cracks of the walls, spaces between ceiling and ceiling, behind the doors and pictures, and in the peridomicile in the vegetation, corrals of domestic animals, caves, among others. The passive search for triatomines was done with traps that use a chicken live bait^53^. The traps were placed in peridomestic palm trees within 20 m of the houses, for an approximate exposure period of 12 h between 18:00 and 6:00 h. The inhabitants were instructed on recognizing the triatomine insects, and on the collection method. A plastic container with paper folded inside and covered with tulle was used in order to store the insects.

A total of 42 bugs were analyzed, including adults and nymphs. For adults, species were identified using morphological keys^54^. Triatomines were kept at 4 °C in individual vials until DNA extraction, handled with gloves, and processed on a bench cleaned with bleach (~ 0.6% sodium hypochlorite) and ethanol 70%. Each insect was dissected in a sterile disposable petri dish using a new scalpel blade each time to prevent cross-contamination.

DNA was extracted from the distal portion of the triatomines’ abdomen using Qiagen DNeasy Blood & Tissue kit following the manufacturer’s instructions, in a dedicated laboratory. DNA samples were stored at − 20 °C until used.

### PCR amplification of selected markers

We amplified specific markers for the simultaneous identification of triatomine species, vertebrate blood meals, *T. cruzi* parasites and gut microbiota as described before^31,32^. Briefly, we amplified a fragment of triatomine Internal Transcribed Spacer (ITS)-2, using primers ITS2TdF (5′-TGGAAATTTTCTGTTGTCCACA-3′) and ITS2Tria (5′-CAGACAATGCCTAGATGCGA3′) or primers ITS2_200F (5′-TCGYATCTAGGCATTGTCTG-3′) and ITS2_200R (5′-CTCGCAGCTACTAAGGGAATCC-3′) for species identification^18,40^. Because these primers fail to consistently amplify ITS2 sequences from Rhodnius species, we also used primers CYTB7432F (5′-GGACG(AT)GG(AT)ATTTATTATGGATC-3′) and CYTB7433R (5′-GC(AT)CCAATTCA(AG) GTTA(AG)TAA-3′)^55^ to amplify a fragment of Cytochrome B for these samples. To identify triatomine blood meal sources, we used two sets of primers targeting the 12S rRNA gene of vertebrates. Similarly, two sets of primers were used to amplify a 140 bp and a 400 bp fragment of the bacterial 16S rRNA gene, respectively, as described previously^56,57^, to characterize the composition of the microbiota. Finally, samples were tested for the presence of *T. cruzi* parasites through amplification of satellite DNA with primers TcZ1/TcZ2. Positive samples were genotyped by PCR amplification of the mini-exon sequence using a multiplex PCR as described by Souto et al., which gives PCR products of different sizes according to the DTU^58^, and newly designed primers that amplify a larger fragment of 500 bp of this marker^59^. PCR reactions were carried out in a C1000 Touch™ Thermal Cycler (Hercules, CA, USA), and PCR products were separated in 2% agarose gels, stained with ethidium bromide and analyzed with Bio-Rad Gel Doc XR+ with Image Lab™ software (Hercules, CA, USA). PCR reaction preparation, PCR apmplification and gel electrophoresis were performed in separate laboratory rooms, to prevent contaminations. In addition, all PCR reactions included positive and negative controls.

### Next generation sequencing

PCR products for blood meal source, bacterial microbiota, *T. cruzi* genotypes and triatomine ITS2 were pooled for each bug and purified for sequencing on a MiSeq (Illumina) platform (paired sequencing). For this, PCR products were end-repaired and indexed/tagged during library preparation to allow for multiplexing of individual bugs for the sequencing. Each bug/library was used to generate 500,000–1,000,000 reads, corresponding to 1000–100,000 reads per marker. Raw Fastq sequences files were imported in Geneious 11.0.2 software for analysis and mapped to each specific DNA marker using reference sequences. Sequences were filtered for quality and length and trimmed of PCR primer sequences and of any other added sequences from the indexing. Raw sequence data are available in NCBI SRA database under BioProject PRJNA701552 (BioSample accession number SAMN17885581–SAMN17885622).

### Sequence and data analysis

Potential sequence chimera were filtered using UCHIME^60^. Sequence variants were then identified using FreeBayes SNP/variant tool^61^, to distinguish between sequencing errors/artifacts and significant sequence variants. For triatomine ITS-2 and CytB sequence analysis, haplotypes were aligned with reference sequences of the species for a precise taxonomic identification of the triatomines. Novel ITS-2 and CytB sequences were deposited in GenBank under accession number MW262950–262967 and MW267836–MW267846.

For blood meal sources, 12S sequences were analyzed by MEGABLAST against the entire “nr” GenBank database. A sequence match with > 97% identity was used for species/genus identification. Sequences were further filtered to remove low abundance sequences (< 0.5%) and normalized using the Total Sum Scaling (TSS) method, to account for variability in sequencing depth among samples. Rarefaction curves were elaborated in Microbiome Analyst^62^, at the individual and group level, to estimate species richness of our sampling of feeding host diversity. Beta diversity of feeding sources among triatomine species was analyzed by Non-Metric Dimensional Scaling (NMDS), based on Bray–Curtis Index distance measures, and statistical significance of differences in feeding hosts among groups was evaluated by permutational MANOVA (PERMANOVA). We also compared the number of host species detected in single triatomines. Data on the proportion of blood-feeding sources and multiple blood meal sources in single bugs were used to construct feeding networks and parasite transmission pathways in Cytoscape 3.8^31,32^. Separate networks were elaborated for *R. prolixus*, *T. dimidiata* and *T. maculata*. Network density, network centralization and network heterogeneity descriptive statistics were calculated as implemented in Cytoscape to summarize network topography and compare networks.

Parasite sequences from the mini-exon gene were first aligned with sequences from reference strains covering all DTUs (TcI: SylvioX10, FcHcl1, Raccoon70; TcII: Tu18, TcIII: M6241; TcIV: CanIII, TcV: SC43; TcVI: CLBrener; TcBat: TCC949cl3), and phylogenetic trees were constructed using the Approximately-Maximum Likelihood method as implemented in FastTree, wich uses the general time-reversible (GTR) model of nucleotide evolution^63^. All mini-exon sequences were deposited in GenBank database under accession numbers MW256825–MW257111. Mini-exon sequences from multiple TcI strains from across the Americas were then used for further comparison (Supplementary Table S1). TcI sequences were analyzed in Beast 2.6.2^64^ and the best model based on Akaike information criteria was based on a HKY substitution model for a constant coalescent population, and the molecular clock was set based on substitution frequency of 7.1 × 10^–8^ as previously established for *T. cruzi*^65^. The model was run for 10 million iterations to obtain the maximum credibility tree. Isolation by distance was tested using Mantel test as implemented in Past3 software^66^ and pair-wise geographic distances among strains were based on their country of origin.

For microbiota composition, bacterial 16S sequences were analyzed using a Bayesian classifier from the Ribosomal Database Project^67^. Taxonomic identification of bacteria was made at a threshold of > 97% sequence identity at the level of bacterial families, Rarefaction curves were elaborated in Microbiome Analyst^62^. Alpha diversity of microbiota communities (within bugs) was assessed by calculating Chao1 and Shannon indexes, which were compared by Student *t*-test or ANOVA, depending on the number of groups compared. Beta diversity (among triatomine species or sub-populations) was compared by Non-Metric Dimensional Scaling (NMDS), based on Bray–Curtis Index distance measures, and statistical significance of differences among groups was evaluated by permutational MANOVA (PERMANOVA). We assessed potential differences among triatomine species, and within species among sex/developmental stage. Finally, correlation networks were elaborated to evaluate potential associations among bacterial families of the microbiota and *T. cruzi* infection, based on Spearman rank correlation. A global network was elaborated, as well as a network for *T. dimidiata* only. All analyses were performed as implemented in Microbiome Analyst software^62^.

## Supplementary Information

Supplementary Figures.

Supplementary Table S1.
