# Supplementary material for: Diversity and interactions among triatomine bugs, their blood feeding sources, gut microbiota and Trypanosoma cruzi in the Sierra Nevada de Santa Marta in Colombia
Source: Sci Rep. 2021 Jun 10;11:12306. doi: 10.1038/s41598-021-91783-2 (PMC8192545; doi:10.1038/s41598-021-91783-2)
Supplement: Supplementary file 1 — Supplementary Figures. [file 41598_2021_91783_MOESM1_ESM.pdf]

Diversity and interactions among triatomine bugs, their blood feeding sources, gut microbiota and *Trypanosoma cruzi* in the Sierra Nevada de Santa Marta, Colombia

Claribel Murillo Solano, Jaime López-Domínguez, Rafael Gongora, Andres Rojas-Gulloso, Jose Usme-Ciro, Erick Perdomo, Claudia Herrera, Gabriel Parra Henao, Eric Dumonteil

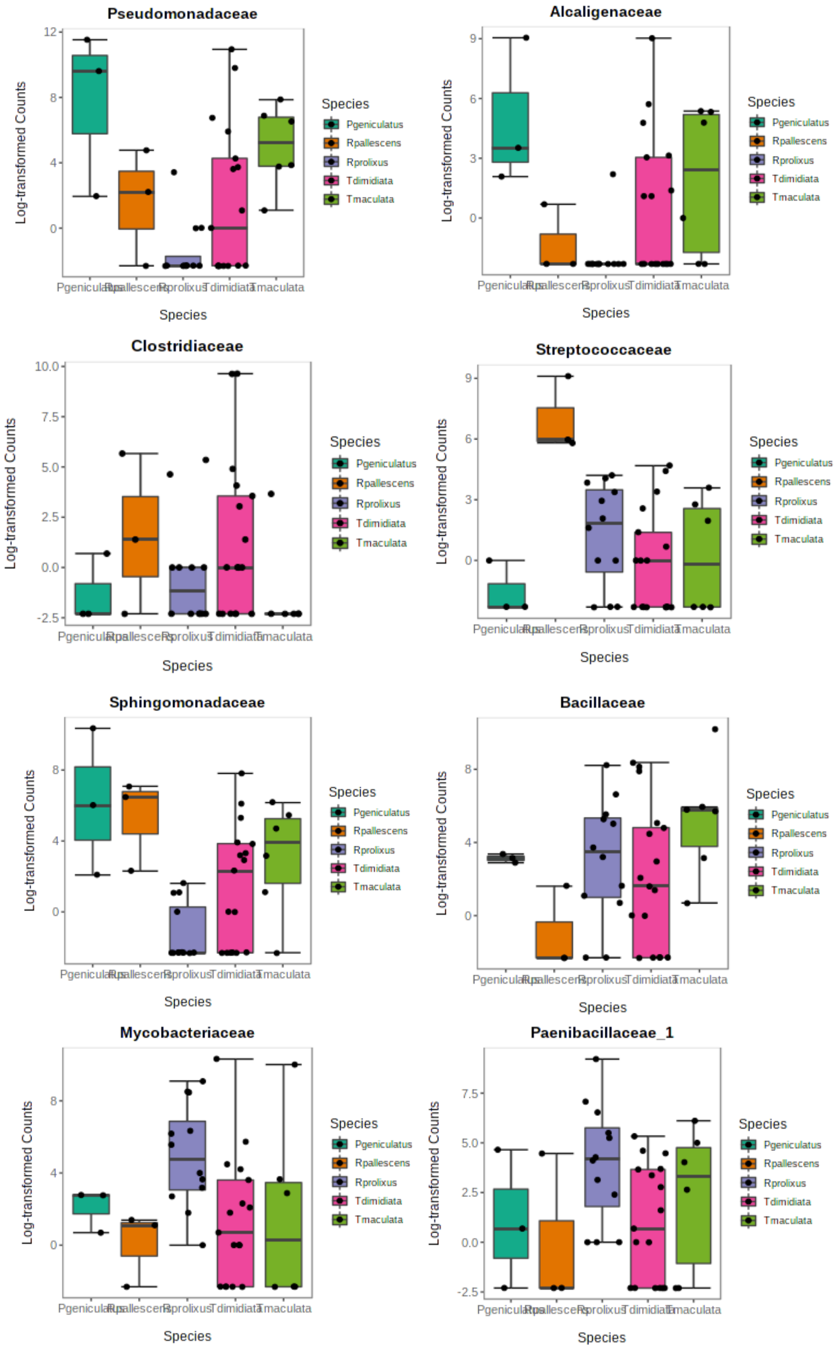

Supplementary Figure S1. Abundance of bacterial families in the microbiota of triatomine species.
Normalized and log-transformed abundance of selected bacterial families are shown for the indicated species of triatomines.

Diversity and interactions among triatomine bugs, their blood feeding sources, gut microbiota and *Trypanosoma cruzi* in the Sierra Nevada de Santa Marta, Colombia

Claribel Murillo Solano, Jaime López-Domínguez, Rafael Gongora, Andres Rojas-Gulloso, Jose Usme-Ciro, Erick Perdomo, Claudia Herrera, Gabriel Parra Henao, Eric Dumonteil

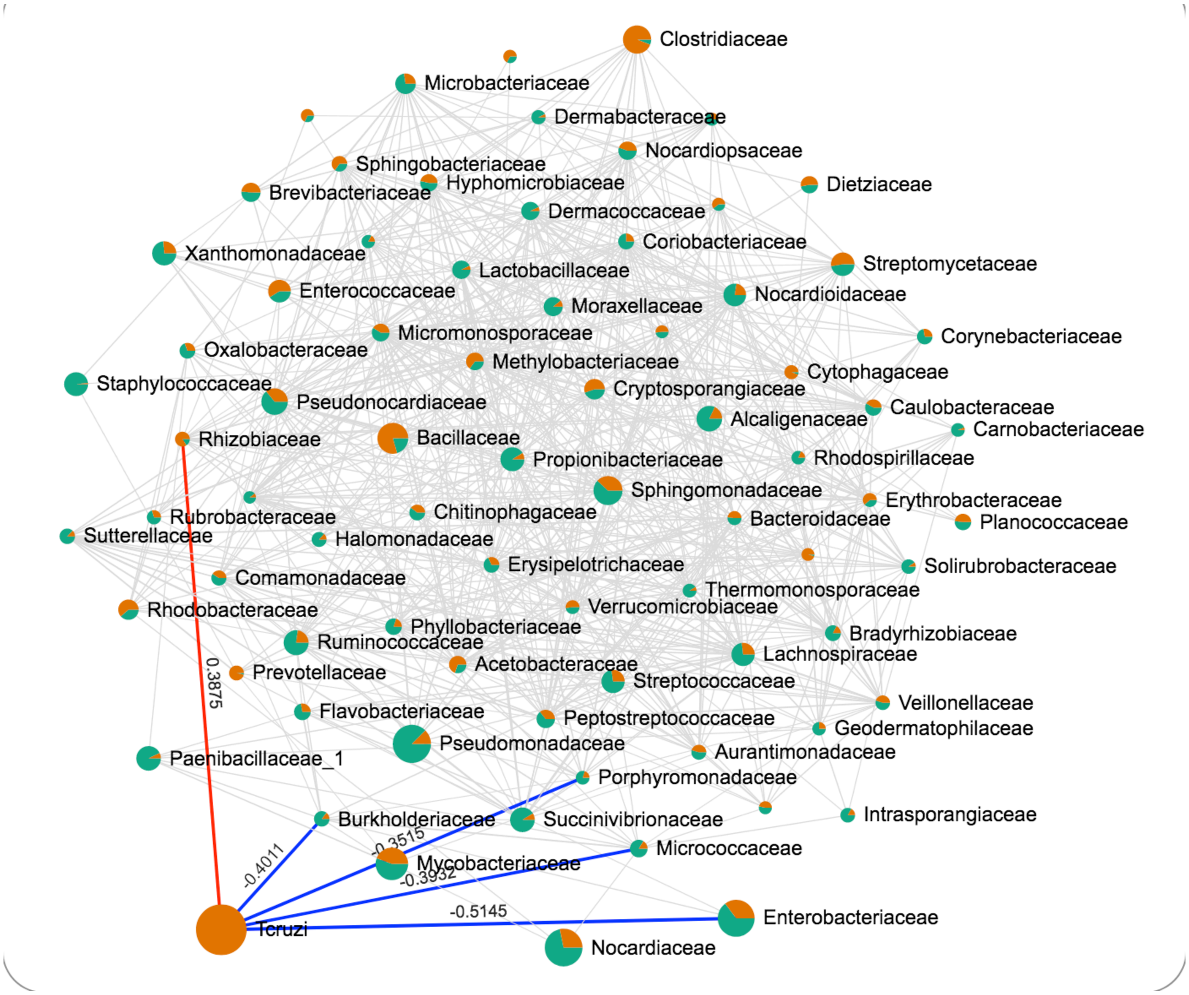

Supplementary Figure S2. Correlation among bacterial families and *T. cruzi* in triatomines
The network illustrates the diversity of bacterial families in triatomine species from the Santa Marta region in Northeastern Colombia, indicated as nodes which size is proportional to the abundance of each family (except for *T. cruzi*). Green nodes indicate bacterial families found in uninfected bugs, and orange nodes indicate those found in *T. cruzi* infected bugs. Edges link nodes/families that are significantly correlated in the bugs (R>0.35). *T. cruzi* infection was positively correlated (red edge) with the presence of Rhizobiaceae (R=0.39), and negatively correlated (blue edges) with the presence of Burkhoderiaceae (R=-0.35), Porphyromonadaceae (R=-0.39), and Enterobacteriaceae (R=-0.51).
